# Comparative Evaluation of Injectable Platelet-Rich Fibrin with and Without Microneedling in Periodontal Regeneration: A Prospective Split-Mouth Clinical Study

**DOI:** 10.3390/biomedicines14010135

**Published:** 2026-01-09

**Authors:** Iulia Muntean, Alexandra Roi, Lavinia Cosmina Ardelean, Laura-Cristina Rusu

**Affiliations:** 1 University Clinic of Oral Pathology, Multidisciplinary Center for Research, Evaluation, Diagnosis and Therapies in Oral Medicine, “Victor Babes” University of Medicine and Pharmacy Timisoara, 2 Eftimie Murgu Sq., 300041 Timisoara, Romania; iulia.sauciur@umft.ro (I.M.); alexandra.moga@umft.ro (A.R.); laura.rusu@umft.ro (L.-C.R.); 2 Academic Department of Technology of Materials and Devices in Dental Medicine, Multidisciplinary Center for Research, Evaluation, Diagnosis and Therapies in Oral Medicine, “Victor Babes” University of Medicine and Pharmacy Timisoara, 2 Eftimie Murgu Sq., 300041 Timisoara, Romania

**Keywords:** periodontitis, microneedling, injectable platelet-rich fibrin, periodontal regeneration, clinical attachment level

## Abstract

**Background/Objectives:** Periodontal disease is a prevalent chronic inflammatory condition that often progresses to irreversible tissue destruction. This study aimed to evaluate the clinical efficacy of a combined minimally invasive periodontal therapeutic protocol scaling and root planing (SRP) with injectable platelet-rich fibrin (i-PRF) and microneedling (MN) compared to conventional SRP with i-PRF alone in patients with stage II–III periodontitis. **Methods:** A prospective split-mouth clinical study was conducted on 54 patients diagnosed according to the 2018 EFP/AAP classification. Each participant received SRP + i-PRF in the upper arch (control) and SRP + i-PRF + MN in the lower arch (test). Periodontal parameters clinical attachment level (CAL), bleeding on probing (BOP), and plaque index (PI) were measured at baseline, 1, 3, and 6 months. Data were analyzed using Friedman and Wilcoxon tests with Bonferroni correction. **Results:** Both treatment protocols produced significant longitudinal improvements in CAL, BOP, and PI (*p* < 0.001). The most pronounced BOP reduction occurred within the first month, while CAL improvement was progressive and stabilized after six months. The Combined protocol achieved slightly greater CAL gain at 6 months (mean difference ≈ 0.46 mm; *p* = 0.0013), indicating a modest yet statistically significant advantage in attachment recovery. Correlation analyses confirmed a coherent healing trajectory characterized by early inflammation resolution, plaque control, and later tissue stabilization. **Conclusions:** Both i-PRF-based regenerative approaches significantly improved periodontal parameters. The addition of MN enhanced CAL recovery and may favor early vascularization and collagen remodeling. Although the clinical difference was limited, the biological plausibility and sustained improvement suggest that MN could represent a valuable adjunct to non-surgical regenerative periodontal therapy. Longer-term studies are warranted to assess the durability of these effects.

## 1. Introduction

Periodontal disease is one of the most prevalent chronic inflammatory conditions, contributing to significant implications for oral health and overall functioning. The current data, from the Global Burden of Disease Study 2021, estimates that approximately 1.07 billion people are being affected [1,2]. Globally, age-standardized prevalence rates for periodontitis increased by approximately 8–10% during the studied period (1990–2021). Projections for the period 2022–2040 indicate a significant increase in incidence (+27.9%), prevalence (+39.7%), and disability-adjusted life years (+38%) [3]. The biggest burden of disease occurs in regions with low sociodemographic index (SDI), with a negative correlation between SDI and age-standardized prevalence [3].

World Health Organization ranks severe periodontal diseases as the fourth most widespread oral condition [4]. Depending on the severity, it is classified in two major forms: gingivitis and periodontitis [5]. Gingivitis is the early stage of periodontal disease in which the gums are characterized by reversible inflammation, without loss of attachment and bone destruction [5]. Untreated, gingivitis can progress to periodontitis, an irreversible condition in which the inflammation destroys supporting tissues such as the periodontal ligament and the adjacent alveolar bone [6]. Differential diagnosis of gingivitis and periodontitis implies the assessment of clinical attachment loss (CAL), probing pocket depth, and radiographic bone loss [7].

According to the American Academy of Periodontology, periodontitis is the primary cause of tooth loss in adults [8]. The literature describes four progressive stages: Stage I (initial), Stage II (moderate), Stage III (advanced), and Stage IV (severe, with extensive attachment loss and functional impairment) [9].

The modern European Federation of Periodontology/American Academy of Periodontology (EFP/AAP) classification of periodontal disease, adopted in 2018 by the World Workshop on the Classification of Periodontal and Peri-Implant Diseases and Conditions, introduced the concepts of staging and grading: the stages (I–IV) reflect the severity and complexity of the disease, while the grades (A–C) indicate the rate of progression and the risk of future periodontal breakdown [10]. The application of this standardized classification improves diagnostic accuracy and allows for a more objective assessment of disease severity and progression [10].

The disease is often underdiagnosed due to nonspecific early symptoms, which favors progression to advanced forms with a major impact on quality of life [11].

Periodontal disease has a multifactorial etiology, being influenced by both modifiable and non-modifiable factors. Modifiable factors include smoking, uncontrolled diabetes, obesity, stress, poor oral hygiene, and use of certain drugs that play a role in expediting of the inflammatory process and loss of periodontal attachment [12,13,14].

Non-modifiable factors such as age, male sex, genetic predisposition, low socio-economic status, osteoporosis, and hormonal changes (puberty, pregnancy, menopause) enhance periodontal disease susceptibility [14].

In young adults, periodontitis is influenced by several risk factors that accelerate the disease progression. The most important are smoking, which negatively affects the immune response and tissue repair, and obesity, which is associated with chronic inflammation induced by adipose tissue. Other predictive elements include gingival bleeding during tooth brushing, low bone mineral density, and low socio-economic status, which contribute to a higher susceptibility to moderate and severe forms of periodontitis [15].

The progressive degradation of the tooth-supporting structures is mediated by the interplay of the subgingival bacterial biofilm and the host immune system, which causes a chronic inflammatory response leading to epithelial attachment destruction, bone resorption, and eventually tooth loss [16].

While the traditional treatment, consisting of scaling and root planing (SRP) is considered as the standard approach for the management of periodontal disease, it is not always sufficient to guarantee complete tissue regeneration and prevent relapses [17]. SRP is mainly indicated in the early and moderate (I and II) stage of periodontitis, when the probing pocket depth is up to 5 mm and clinical attachment loss is moderate [17,18]. In these stages, SRP is intended to eliminate bacterial plaque and subgingival calculus, to decrease inflammation, and support tissue reattachment. For advanced stages (III and IV), characterized by deeper pockets (≥6 mm) and extended bone loss, the efficacy of SRP diminishes, and surgical periodontal therapies are required [18]. However, SRP constitutes an essential first phase, which acts by reducing bacterial load and assess tissue response, before undergoing more complex interventions [19].

Lately, the interest in minimally invasive periodontal regenerative therapies has increased. Microneedling (MN), or collagen induction therapy, a promising treatment method, uses micro-needles to generate controlled injuries in the gingiva and stimulate tissue healing by collagen synthesis and release of growth factors [20,21]. Although the use of MN as an adjuvant minimally invasive therapy that stimulates the local production of collagen has become a popular approach for targeting the regeneration of the derma [22], the gingiva may also benefit from this procedure. By inducing minor superficial bleeding points, the healing process is stimulated and the regenerative process is activated.

Platelet-Rich Fibrin (PRF) has been used in dentistry for more than a decade as a tissue regeneration method, by slow release of growth factors, promoting neovascularization and tissue remodeling [23]. The injectable form, i-PRF, developed by slow centrifugation, provides an autologous liquid suspension with superior regenerative effects. PRF, especially i-PRF, contains a high concentration of platelets and leukocytes, thus releasing growth factors (PDGF, TGF-β, VEGF) as well as promoting angiogenesis, fibroblast proliferation, and collagen remodeling [24,25,26]. The integration of PRF in dentistry represents an important asset for the clinical outcome of various surgical interventions, being an important factor in the guided healing process [27].

When combined with i-PRF, MN, which stimulates the local release of healing factors and promotes collagen genesis, produces a synergistic effect [28].

**Figure 1:**
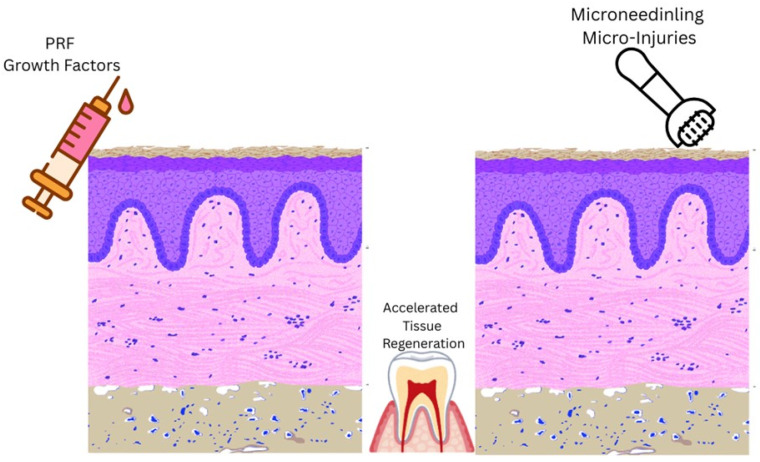
Schematic representation of the mechanism of action of combined i-PRF and MN therapy.

The mechanism of action of PRF and MN therapy is depicted in Figure 1.

The association of these two approaches as clinical treatments for various oral pathologies has not been yet intensively studied. However, the associating of their local application may become an important step towards new treatment approaches, as to date, studies reveal the improvement of the gingival phenotype by increasing of the collagen production [29]. These findings represent an important start-point for the present study, which explores the probability of healing stimulation, neovascularization, and periodontal regeneration.

The aim of this prospective split-mouth clinical study is to evaluate the clinical efficacy of a combined periodontal therapeutic protocol, which includes SRP followed by the application of MN and i-PRF, compared with SRP and i-PRF application, in patients with stage II or III periodontitis, according to the EFP/AAP classification.

The specific objectives of the present study are as follows:

1. To evaluate the efficacy of SRP combined with MN and the association of i-PRF in patients diagnosed with stage II and III periodontitis.
2. To perform a comparative analysis of the effectiveness of the SRP + MN + i-PRF protocol compared to using SRP + i-PRF alone.
3. To determine the clinical benefits of using the additional MN technique, in association with SRP and i-PRF.

In order to determine the adjuvant effect of the combined periodontal therapeutic protocol on reducing gingival inflammation the bleeding on probing (BOP), and gingival edema were monitored. The stability of the clinical outcome was analyzed by follow-ups at 1, 3 and 6 months.

## 2. Materials and Methods

### 2.1. Cohort and Study Design

This prospective split-mouth clinical study included 54 patients diagnosed with stage II or III periodontitis according to the EFP/AAP classification. The study was conducted at the “Victor Babeș” University of Medicine and Pharmacy in Timișoara, within the Department of Oral Pathology, between March 2024 and September 2024. The study protocol was approved by Research Ethics Committee of Victor Babeș University of Medicine and Pharmacy, Timișoara, approval number 81/10.09.2021 rev 2024, and conducted in accordance with the principles of the Declaration of Helsinki. All patients signed an informed consent prior to inclusion in the study. The upper dental arch was treated with SRP followed by i-PRF application (Standard protocol), while the lower dental arch underwent SRP combined with MN and i-PRF (Combined protocol). Each patient received both types of treatment. The reason of selecting this treatment protocol was to evaluate the potential additional regenerative effect of MN when associated with i-PRF, compared to the standard SRP + i-PRF protocol. The upper arch served as control, as it was treated using the conventional non-surgical regenerative approach using i-PRF after SRP, while the lower arch acted as the test, as MN was integrated to stimulate local microcirculation, enhance growth factor release, and increase i-PRF penetration through the gingival tissue.

All clinical procedures, including diagnosis, grading, periodontal assessment, SRP, i-PRF preparation and application, as well as MN, were performed by a single clinician to minimize inter-operator variability. Therefore, inter-operator calibration was not necessary.

The split-mouth model enabled direct intra-patient comparison under identical systemic, microbiological, and environmental conditions, thus minimizing inter-individual variability and increasing the reliability of the obtained results. From a clinical standpoint, the choice of applying MN to the lower dental arch was influenced by the initial probing depth values. The lower arch sites generally presented deeper periodontal pockets and more pronounced inflammatory changes compared to the upper ones, thus requiring an enhanced regenerative stimulus. MN was therefore introduced as an adjunctive technique to improve tissue perfusion, stimulate fibroblast activity, and facilitate deeper penetration of growth factors from i-PRF into periodontal structures. The upper dental arch, which exhibited shallower probing depths and milder inflammatory status, was treated with conventional SRP combined with i-PRF, serving as control for evaluating the additional benefits of MN in sites with more advanced periodontal destruction.

Inclusion and Exclusion Criteria

The study included patients diagnosed with stage II or III, grade A, B periodontitis according to the EFP/AAP classification. Eligible subjects presented periodontal pockets ≥4 mm after professional oral cleaning. No periodontal treatment was performed within the last 6 months and had no systemic diseases that might affect healing (e.g., uncontrolled diabetes mellitus or severe autoimmune disorders). Exclusion criteria included active smoking (>5 cigarettes/day), stage IV periodontitis, and poor oral hygiene upon initial cleaning.

The analyzed cohort (Table 1) consisted of 54 patients, including 28 females (51.85%) and 26 males (48.15%), with mean age of 43.7 ± 8.1 years (range: 29–62 years). In terms of age distribution, 17 participants (31.48%) were aged under 40 years, 36 (66.67%) were aged between 40 and 59 years, and 1 (1.85%) was aged ≥60 years. A total of 46 participants (85.19%) lived in urban areas, while 8 subjects (14.81%) were rural residents. Concerning lifestyle habits, 11 participants (20.37%) reported smoking ≤ 4 cigarettes per day, whereas 43 (79.63%) were non-smokers.

G*Power software (version 3.1.9.7; Heinrich Heine University Düsseldorf, Düsseldorf, Germany) was used to conduct a post hoc power analysis for a paired-samples design. An effect size of Cohen’s dz = 0.49 was determined (mean difference = 0.463 mm; SD of paired differences = 0.946 mm). The study was sufficiently powered to identify the observed inter-protocol difference, as evidenced by the achieved statistical power (1 − β) of 0.94 with a two-tailed α error probability of 0.05 and a total sample size of 54 paired observations.

### 2.2. Assessment and Therapeutic Protocol

At the first visit, each patient underwent a complete anamnesis and clinical evaluation, followed by supra-gingival scaling and oral hygiene instruction, including tooth brushing and interproximal cleaning techniques. A week after the first visit (baseline), a detailed periodontal evaluation was carried out. SRP was performed at all sites exhibiting periodontal pockets, followed by the application of i-PRF or MN + i-PRF, depending on the protocol. The periodontal status was re-evaluated at 1, 3, and 6 months to assess clinical improvements and healing progression. The following parameters were determined: periodontal pocket depth (PPD), bleeding on probing (BOP), plaque index (PI), and clinical attachment loss (CAL).

**Figure 2:**
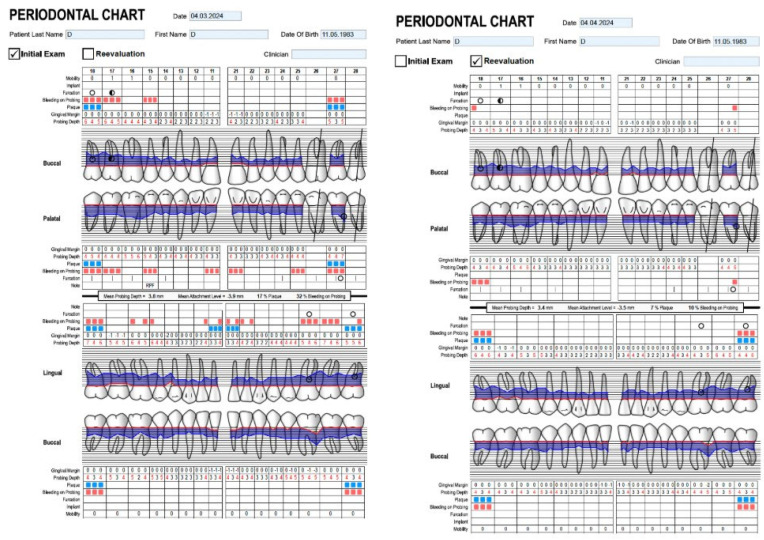
The digital periodontal chart.

The measurements were identically performed for both groups using a Perioscreen Colorvue periodontal probe (Hu-Friedy Manufacturing Co., Chicago, IL, USA; gradated at 3–5–7–10 mm), by a dentist with extensive experience in periodontal screening. PPD was measured in millimeters, from the gingival margin to the most apical point of the sulcus/pocket, at six sites per tooth, to the nearest millimeter: mesiobuccal, buccal, distobuccal, mesiolingual, lingual, and distolingual. The gingival margin was also measured at the same sites, and CAL was calculated for all locations, as the distance from the cemento-enamel junction to the base of the periodontal pocket. Each patient’s measurements were recorded in a digital periodontal chart (Periodontal Chart Online, Figure 2).

**Figure 3:**
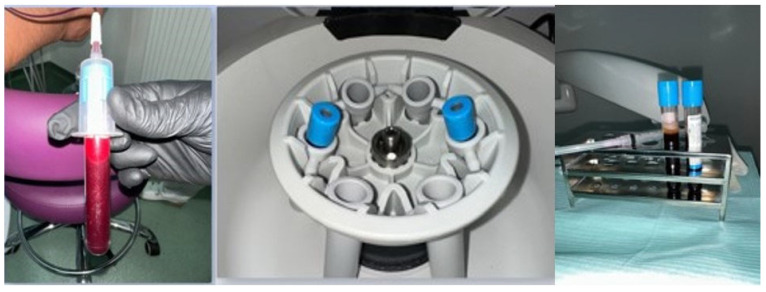
i-PRF preparation.

**Figure 4:**
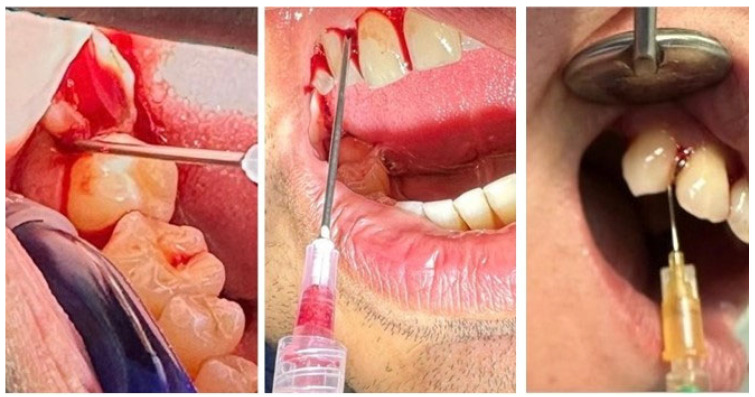
i-PRF administration.

**Figure 5:**
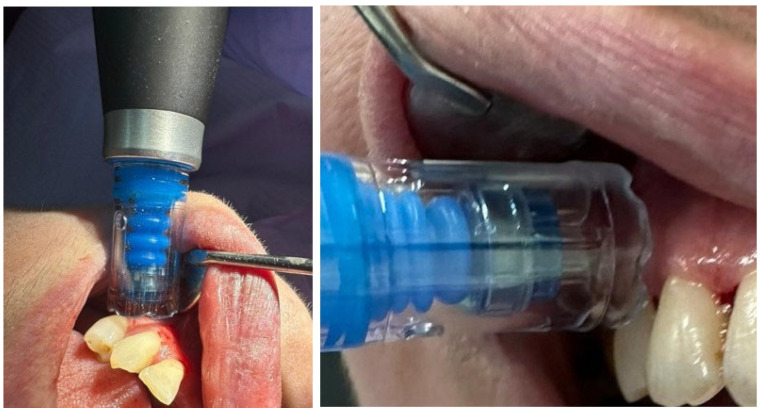
MN procedure.

BOP was recorded 10 s after probing and considered positive or negative depending on the presence or absence of bleeding at the probed site, and expressed as a percentage of positive sites. PI was assessed by visual inspection and scored according to the Silness-Löe index (0–3). SRP was performed using an ultrasonic scaler with subgingival tips (EMS Piezon, Electro Medical Systems SA, Nyon, Switzerland) and manual Gracey curettes (#1–2, #7–8, #11–12, #13–14; Hu-Friedy Manufacturing Co., Chicago, IL, USA). Physiological saline was used for irrigation, and a 0.2% chlorhexidine solution (Curasept, Curaden, Kriens, Switzerland) was used as an antiseptic. For i-PRF preparation, venous blood was collected using sterile vacutainers and needles (BD Vacutainer, Becton Dickinson, Franklin Lakes, NJ, USA), and centrifuged in a PRF centrifuge (Process, PRF Duo, Nice, France) at 700 rpm for 3 min (Figure 3). RCF, a standardized parameter that ensures the application of the same centrifugation conditions, has been calculated according to the formula: RCF (× *g*) = 1.118 × 10^−5^ × r (rotor radius) × (rpm)^2^. The rotor radius for Process PRF Duo centrifuge (Nice, France) is ≈11 cm, resulting in a RCF value of ≈60 g (1.118 × 10^−5^ × 11 × (700)^2^). The resulting i-PRF was collected with sterile pipettes and applied to the treated sites, which were then protected with sterile dressings (Figure 4). MN was performed using a Dermapen device (DermapenWorld, Sydney, Australia) equipped with disposable sterile needle cartridges (adjustable depth 0.25–2.5 mm) (Figure 5). A 0.12% chlorhexidine topical antiseptic was applied before the procedure, and sterile gauze pads were used for hemostasis and post-procedure care.

### 2.3. Data Collection

The longitudinal clinical data recordings for the 54 patients were stored in a master Excel file. Patient characteristics were retrieved from the baseline demographic information (sex, age, age group) and risk/context variables (smoking: yes/no; environment: urban/rural) database. The primary clinical outcome, CAL was captured at four time points: baseline, 1 month, 3 months, and 6 months, and was also recorded in an Excel file using two assessment approaches: Standard and Combined (columns: “CAL Initial Standard/Combined”, “CAL 1 month Standard/Combined”, “CAL 3 months Standard/Combined”, “CAL 6 months Standard/Combined”). Two complementary Excel files, providing BOP and PI for the same cohort were linked by patient identifier and time point to provide context on CAL changes and to facilitate subgroup and covariate analysis (e.g., by smoking status, environment, or age group).

### 2.4. Statistical Analysis

Statistical analysis was performed using the JASP statistical software, version 0.18 (JASP Team, University of Amsterdam, Amsterdam, The Netherlands) and StatsKingdom online statistical software (StatsKingdom, Melbourne, Australia).

The longitudinal evolution of periodontal parameters (CAL, BOP, and PI) was analyzed using a nonparametric repeated-measures approach. Because of the correlated nature of repeated observations for each subject, the Friedman test was applied to assess overall temporal differences within each treatment protocol (Standard and Combined). This nonparametric method was selected based on several diagnostic criteria: the absence of normal distribution for paired differences, confirmed by Shapiro–Wilk tests (*p* < 0.05 for all variables), and the presence of within-subject dependence inherent to longitudinal periodontal data. When the Friedman test indicated significant temporal variation, post hoc pairwise Wilcoxon signed-rank tests were conducted to identify specific time intervals responsible for the observed differences (e.g., baseline–1 month, 3–6 months). To control the risk of Type I error due to multiple comparisons, Bonferroni correction was applied, and adjusted *p*-values < 0.05 were considered statistically significant. As each patient received both types of treatment (split-mouth design), in order to compare the two treatment protocols: Standard (SRP + i-PRF) and Combined (SRP + i-PRF + MN), the Wilcoxon signed-rank test for paired samples was performed at each follow-up interval. This test was preferred over the paired *t*-test due to the non-normality of the data and the ordinal nature of several clinical indexes. Effect sizes were computed using the formula r = Z/√N and interpreted as small (0.1–0.3), moderate (0.3–0.5), or large (>0.5). Correlations between CAL, BOP, and PI were assessed using Spearman’s rank correlation coefficient (ρ), providing insight into the dynamic relationships between plaque accumulation, inflammation, and attachment gain throughout the healing period. Finally, a subgroup analysis, to explore potential impact of sex, age group, smoking status, and living area (urban/rural) on BOP and PI was conducted using the Mann–Whitney U test, given the independent and non-normally distributed nature of these categorical comparisons.

## 3. Results

### 3.1. Sex Distribution Across Age Groups

**Figure 6:**
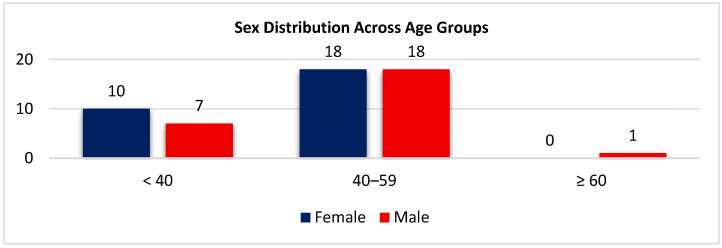
Distribution by sex across age groups (<40, 40–59, ≥60 years).

As shown in Figure 6, middle-aged adults predominate in both sex groups. Among females, 10 (35.7%) were <40 years old and 18 (64.3%) were 40–59 years old. Among males, 7 (26.9%) were <40 years old, 18 (69.2%) were 40–59 years old, and 1 (3.9%) was ≥60 years old. Overall, the age distribution was comparable between sexes, with a clear concentration in the 40–59-year group. Both sexes were similarly represented in the 40–59-year category, with a lower proportion of participants younger than 40 and only one male aged ≥60.

### 3.2. Sex Distribution by Environment

**Figure 7:**
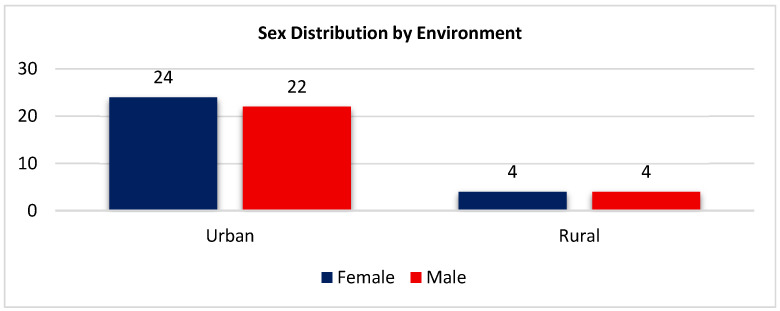
Comparison of male and female participants according to environment (urban vs. rural).

More than 85% of the participants were urban residents. Among females, 24 (85.7%) lived in the urban area and 4 (14.3%) in rural settings, while among males, 22 (84.6%) resided in the urban area and 4 (15.4%) in rural environments. This indicates a broadly homogeneous geographical distribution in the sex groups, with prevailing urban residency (Figure 7).

### 3.3. Age Group Distribution by Environment

**Figure 8:**
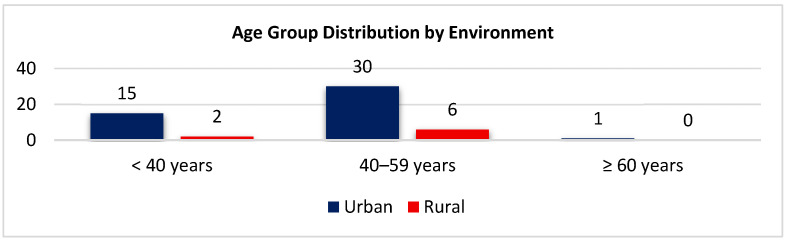
Distribution of age groups by environment.

Most participants, across all age categories, were urban residents (Figure 8). Among the under 40-years group, 15 (88.2%) lived in urban areas and 2 (11.8%) in rural areas. Within the 40–59-year group, 30 (83.3%) were urban residents and 6 (16.7%) were rural inhabitants. The single participant aged ≥ 60 years lived in an urban environment. This distribution confirms the predominance of urban residency irrespective of age.

The demographic composition of the 54-patient cohort is consistent with a balanced sex ratio and a predominantly middle-aged, urban, non-smoking population.

The homogeneous nature of both age and environment distribution strengthens the internal consistency of later analyses of treatment-related changes in CAL, BOP, and PI.

### 3.4. Indicator Measurements

#### 3.4.1. CAL—Clinical Attachment Level

At each time point (baseline, 1 month, 3 months, and 6 months), CAL was recorded for both Standard (SRP + i-PRF) and Combined (SRP + i-PRF + MN) protocols (Table 2). Mean (x), the arithmetic average of CAL values, indicates the general trend of the group. Median is the middle value of the dataset, less influenced by outlier values. Q1 (first quartile), the value below which 25% of observations fall, represents the lower limit of the central distribution. Q3 (third quartile) is the upper limit of the central distribution, the value below which 75% of observations fall. The interquartile range (IQR = Q3-Q1) includes 50% of the central values and reflects normal data variability with little change following high outliers. These indicators offer a detailed snapshot of overall bias and distribution of clinical outcomes among patients.

**Figure 9:**
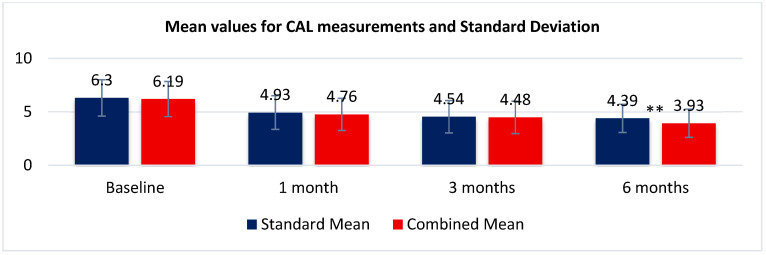
Mean values of CAL at baseline, 1 month, 3 months, and 6 months for both treatment protocols. Error bars represent standard deviation (mean ± SD). Statistical significance between protocols at the same time point is indicated by asterisks (** *p* < 0.01).

The means (6.30 mm) and medians (both 6 mm) of the Standard and Combined protocols were comparatively similar at baseline, confirming the similarity of the initial periodontal conditions. The interquartile ranges (2–3 mm) indicate moderate variability across subjects, with only a few high-CAL outliers (maximum = 10 mm). By 1 month, mean CAL decreased markedly in both groups (Standard = 4.93 mm; Combined = 4.76 mm), representing an initial reduction of approximately 1.4 mm and 1.5 mm, respectively. This early improvement corresponds to the inflammatory-resolution phase and rapid soft-tissue remodeling. At 3 months, the trend was the same, showing additional marginal reduction (Standard = 4.54 mm; Combined = 4.48 mm). The dispersion values remained stable, indicating homogeneous responses within the cohort. At 6 months, both groups reached a plateau phase with sustained improvement (Standard = 4.35 mm; Combined = 4.30 mm). The Combined protocol consistently yielded slightly lower CAL values, reflecting a small yet potentially meaningful enhancement in clinical attachment gain (≈0.05 mm difference). Overall, descriptive statistics show a steady improvement of CAL across time for both treatment protocols, following parallel healing trajectories (Figure 9). The differences between protocols were minimal; however, the Combined group’s marginally better values suggest an incremental benefit that may become more pronounced in extended (≥12 month) follow-ups.

##### Paired Comparison: Combined vs. Standard at Each Time Point

To determine whether the addition of MN to the conventional i-PRF-based therapy produced different CAL outcomes, paired comparisons between the SRP + i-PRF and SRP+ i-PRF + MN protocols were conducted at baseline, 1 month, 3 months, and 6 months. Normality testing using Shapiro–Wilk revealed a non-normal distribution of paired differences (*p* < 0.01 in all intervals), supporting the use of a non-parametric approach, the Wilcoxon signed-rank test.

At baseline, results of the Wilcoxon Signed-Rank test indicated no statistically significant difference in CAL between the Standard (Mdn = 6, *n* = 54) and Combined (Mdn = 6, *n* = 54) protocols, Z = −0.92, *p* = 0.356, r = −0.16. Ties were present, so the normal approximation with ties correction was used (non-zero pairs = 35; W = 261; W^−^ = 369, W^+^ = 261; C_ties = 372.75; S.E. = 57.92). The common-language effect size was ~0.41 (probability that a random Standard value exceeds a random Combined value). The analysis included 30 non-zero pairs (ties excluded), with a Wilcoxon statistic W = 194 (W^+^ = 271, W^−^ = 194). This initial statistical equivalence establishes a valid foundation for subsequent within-group comparisons and temporal analyses, ensuring that any later changes can be attributed to treatment effects rather than baseline bias.

At 1 month, the Wilcoxon signed-rank test indicated no statistically significant difference in CAL between the Standard (Mdn = 5, *n* = 54) and Combined (Mdn = 5, *n* = 54) protocols, Z = −0.87, *p* = 0.383, r = −0.15. Ties were present, so the normal approximation with ties correction was used (non-zero pairs = 34; W = 248; W^−^ = 347, W^+^ = 248; C_ties = 268.125; S.E. = 56.15). The mean paired difference (Combined − Standard) was −0.17 (SD = 1.19), suggesting a small, non-significant tendency toward lower CAL for the Combined arm at 1 month. The common-language effect size was ~0.42 (probability that a random Standard value exceeds a random Combined value). Early postoperative healing appears comparable between protocols, with a slight but non-significant numerical advantage for the Combined approach. At 1 month, both treatment protocols exhibited comparable CAL values, with no statistically significant difference. The small negative median ΔCAL (which represents the median value of the differences between the CAL measurements obtained for the two treatment approaches, expressing the typical change in periodontal attachment between the protocols) suggests a minimal numerical advantage for the Combined method, yet the effect size and *p*-value confirm that this variation is not clinically or statistically meaningful. Clinically, these findings indicate that early healing dynamics and soft-tissue adaptation progress similarly in both groups.

At the 3-month evaluation, results of the Wilcoxon signed-rank test indicated a non-significant, very small difference between the Standard (Mdn = 4 mm, *n* = 54) and Combined (Mdn = 4 mm, *n* = 54) protocols, Z = −0.25, *p* = 0.803, r = −0.04. Because ties were present, the normal approximation with ties correction was applied (non-zero pairs = 35; W = 300; W^−^ = 330, W^+^ = 300; ties correction = 335.625; S.E. = 58.24). The mean paired difference (Combined-Standard) was −0.056 ± 1.188 mm, indicating a small and non-significant deviation at this time point. At 3 months, both protocols exhibited essentially identical CAL, with no statistically or clinically meaningful difference. The very small effect size (|r| ≈ 0.04) and a median paired difference of 0 mm confirm that the addition of MN did not alter mid-term attachment outcomes beyond those achieved with i-PRF alone. Clinically, this aligns with a healing stabilization phase (after the early remodeling seen at 1 month), in which soft-tissue maturation shows similar progress.

At the 6-month follow-up, results of the Wilcoxon signed-rank test indicated a significant, large difference between the Standard (Mdn = 4 mm, *n* = 54) and Combined (Mdn = 4 mm, *n* = 54) protocols, Z = −3.21, *p* = 0.0013, r = −0.59. With ties present, the normal approximation with ties correction was used (non-zero pairs = 30; W = 83.5; W^−^ = 381.5, W^+^ = 83.5; ties correction = 228.375; S.E. = 46.21). The mean paired difference (Combined-Standard) was −0.463 ± 0.946 mm, indicating lower CAL values (i.e., better attachment) for the Combined protocol. At 6 months, the Combined protocol achieved statistically better CAL than Standard, with a large rank-based effect (|r| ≈ 0.59) and an average paired advantage of about 0.46 mm. While the absolute value lies under 1 mm (clinically modest), its consistency across pairs and the strength of the rank effect indicate an emerging clinical divergence in favor of the Combined approach. These findings align with the notion that adjunctive MN may enhance later-phase tissue maturation and stability beyond the early remodeling stage. Continued observation at 12–24 months will clarify durability, potential widening of the differences, and clinical relevance for long-term periodontal stability.

##### Longitudinal Change Within Each Protocol

To evaluate the intra-group evolution of periodontal healing over time, longitudinal analyses were performed for each treatment protocol (Standard vs. Combined). The CAL values at baseline, 1 month, 3 months, and 6 months were compared using non-parametric methods due to deviation from normality (as indicated by prior Shapiro–Wilk testing). The Friedman test was used to determine whether significant temporal differences existed within each group, followed by pairwise Wilcoxon signed-rank post hoc comparisons with Bonferroni correction to identify specific time intervals contributing to the overall effect.

Within the Standard protocol, the CAL evolution followed a biphasic pattern: an initial transient rise caused by postoperative edema, followed by a clear and significant reduction between 3 and 6 months, confirming that i-PRF-assisted SRP supports favorable long-term periodontal healing and stable attachment gain.

The Combined protocol exhibited an almost identical healing trajectory: an early, mild increase due to postoperative remodeling, followed by a consistent reduction in CAL during the subsequent months. At the 6-month mark, the large effect size (r ≈ 0.56) indicated a clinically meaningful attachment gain. Although the inclusion of MN produced slightly lower median CAL values at 6 months (3.93 mm vs. 4.39 mm), this difference was not significant in between-group analysis.

Both Standard and Combined protocols showed statistically significant longitudinal CAL improvements across the 6-month period (*p* < 0.001). In both protocols, the main attachment gain occurred between 3 and 6 months, indicating inflammatory resolution and functional tissue integration. While MN did not produce a statistically superior outcome, the healing pattern and timing suggest that i-PRF is predominantly responsible for promoting periodontal regeneration. From a clinical point of view, these results validate i-PRF as a reliable regenerative adjunct, while MN may offer minor, non-significant reinforcement of the reparative process.

#### 3.4.2. BOP-Bleeding on Probing

At baseline, the mean BOP index was 0.38 ± 0.16, reflecting moderate gingival inflammation across the cohort. Following treatment, there was a pronounced and fast decrease to 0.20 ± 0.11 at 1 month, corresponding to a nearly 50% reduction in mean BOP values. The median also dropped from 0.38 to 0.17, suggesting improvement for most patients. At 3 months, the mean BOP showed a slightly decrease to 0.17 ± 0.10, with a median of 0.14, indicating further stabilization of periodontal health and continued enhancement of the soft tissue response. At 6 months, the mean (0.17 ± 0.09) and median (0.17) values remained low, which indicates sustained control of gingival inflammation and stable tissue healing. The BOP values progressively dropped from 0.65 at baseline to 0.33 at 6 months, reflecting a decreasing variability and a homogeneous clinical response within the cohort. These results suggest that both treatment protocols led to a significant and sustained reduction in gingival bleeding. The greatest improvement occurred within the first month, and was retained during the subsequent stages.

##### Longitudinal Change in BOP

To assess the temporal evolution of gingival inflammation, the BOP index was analyzed at each time point: baseline, 1 month, 3 months, and 6 months, for the entire patient cohort (*n* = 54). As BOP data deviated from normal distribution (Shapiro–Wilk *p* < 0.001), the Friedman test was applied to evaluate within-subject changes over time. The analysis demonstrates a statistically and clinically significant reduction in gingival bleeding following treatment, which confirms the efficacy in reducing inflammation. The most significant improvement was observed within the first month, reflecting a fast soft-tissue response and healing. At 3 to 6 months, BOP values stabilized, indicating steady tissue maturation and vascular remodeling. The lack of further changes after 3 months emphasizes the stability of periodontal recovery and the sustained inflammation control (Table 3).

#### 3.4.3. PI-Plaque Index

**Figure 10:**
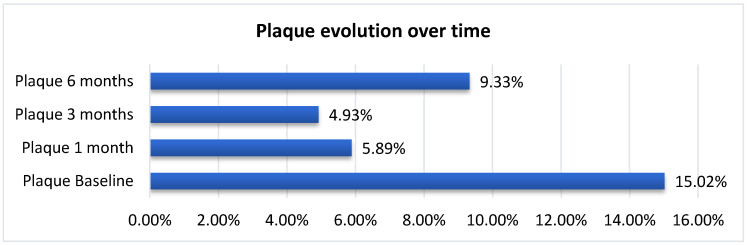
Mean PI evolution over time (baseline, 1 month, 3 months, and 6 months).

PI was also record ed at baseline, 1 month, 3 months, and 6 months to quantify oral hygiene and biofilm accumulation (Figure 10). PI values were expressed as the proportion of sites with visible plaque relative to the total examined sites per patient. At baseline, the mean PI value of 0.15 ± 0.15 (median = 0.13, range: 0.00–0.70), indicates an overall satisfactory oral hygiene across the cohort (*n* = 54), only few patients presenting moderate plaque deposits. The IQR (0.06–0.23) showed moderate variability, primarily reflecting individual hygiene habits and smoking status. At 1 month, the mean PI decreased sharply to 0.06 ± 0.07 (median = 0.05), which corresponds to a 60% decrease, compared to baseline. This marked enhancement demonstrates effective plaque control during the early treatment phase, consistent with improved oral hygiene following instruction and patient compliance after initial SRP and i-PRF. At 3 months, the mean PI remained stable at 0.05 ± 0.06 (median = 0.05), confirming that most patients maintained optimal plaque control. The narrow IQR (0.03–0.08) indicates a homogeneous response across the group. At 6 months, the mean PI value of 0.09 ± 0.07 (median = 0.08), shows a minor increase compared to the value recorded at 3 months. This small variation has no clinical significance and reflects the normal fluctuations in hygiene behavior. The overall PI range narrowed progressively from 0.70 at baseline to 0.30 at 6 months, which indicates decreased inter-individual variability and improved hygiene over time.

The apparent longitudinal decrease in PI demonstrates that both treatment protocols resulted in improved oral hygiene and reduced bacterial biofilm accumulation. The marked initial decline corresponds to early patient motivation and strict compliance after professional intervention. Subsequent stability from 1 to 6 months reflects long-lasting compliance to hygiene instruction, suggesting successful periodontal maintenance.

Overall, these findings suggest that integrating i-PRF therapy within a well-structured hygiene routine results in steady improvement of plaque control—a critical determinant of long-term periodontal health.

##### Correlations Between PI and Demographic or Behavioral Variables

Plaque accumulation decreased significantly and uniformly across all demographic subgroups, confirming that both protocols effectively reinforced oral hygiene. Smoking remained the only factor associated with slightly higher plaque levels, consistent with literature describing tobacco’s negative impact on oral microbial balance [30]. No significant influence was observed for sex, age, or environment, indicating that behavioral compliance and patient motivation were the primary determinants of plaque control success.

#### 3.4.4. Correlation Analysis Between CAL, BOP, and PI

To explore periodontal health and healing, correlation analyses between clinical parameters CAL, BOP, and PI were carried out at each time point. These variables reflect the progression of periodontal recovery, from inflammation and plaque accumulation to soft tissue reattachment and stabilization, their interdependence being an essential indicator of overall treatment success. As all variables deviate from normal distribution (Shapiro–Wilk *p* < 0.05), Spearman’s rank correlation coefficient (ρ) was used. Analyses were performed on the entire cohort (*n* = 54), combining data from both treatment protocols, as there were no significant inter-protocol differences. All correlations were computed on aligned datasets, excluding cases with missing paired data for a given time point.

The combined correlation pattern between CAL, BOP, and PI reveals a constant course of periodontal healing:

1. Early phase (baseline-1 month): the strong interdependence between all three parameters reflects active inflammation, plaque-driven tissue breakdown, and early reparative activity.
2. Intermediate phase (1–3 months): As plaque control and inflammation improved, correlations weakened. Gingival bleeding became less plaque-dependent, and attachment levels began to stabilize.
3. Late phase (6 months): All correlations decrease to weak or non-significant values, indicating a mature tissue stabilization and independence of CAL from surface plaque and bleeding parameters.

Clinically, this pattern demonstrates that while plaque and inflammation control are crucial during the initial healing phase, long-term attachment stability depends primarily on regenerative and host-modulated processes rather than mechanical plaque removal. The sustained low BOP and PI values confirm the long-term effectiveness of both protocols in achieving stable periodontal health.

#### 3.4.5. Comparative Dynamics Between Standard and Combined Protocols

To evaluate the overall outcome of the two treatment protocols, the evolution of CAL, BOP, and PI was compared at each time point. While individual pairwise tests revealed no statistically significant inter-protocol differences at isolated time points, analyzing the global trend provides insight into healing kinetics and clinical stability over time.

When analyzed together, the three clinical parameters (CAL, BOP, PI) define a clear and biologically consistent pattern:

1. Early phase (baseline–1 month): both groups displayed transient CAL elevation and rapid BOP/PI reduction, reflecting early inflammation resolution and adherence to hygiene practice.
2. Intermediate phase (1–3 months): tissue maturation and early reattachment took place. The statistically insignificant differences between Standard and Combined protocols point out to comparable healing kinetics.
3. Late phase (3–6 months): both treatment protocols maintained significant CAL gains and stable periodontal indices. However, the Combined protocol showed a numerically greater CAL improvement (mean difference ≈ 0.4 mm) and a narrower variability range, suggesting more homogeneous tissue integration and enhanced maturation of the regenerated periodontal tissues.

Although the difference did not reach a strong statistical significance at 6 months, the clinical trend favored the Combined technique, indicating that MN may accelerate early cellular responses and promote sustained collagen remodeling. These results justify future extending of the follow-up to 1 year and 2 years. Future evaluation may capture late remodeling occurrence and reveal whether the Combined protocol offers a long-term regenerative advantage besides the short-term equivalent outcome.

## 4. Discussion

The MN technique, initially used in dermatology for skin regeneration and collagen induction, consists of creating controlled micro-injuries that trigger the natural healing cascade (release of growth factors, fibroblast migration, neo-collagen genesis, and neovascularization) [28]. Through these created micro-channels, topical substances with anti-inflammatory and antimicrobial effects can be administered, increasing their local efficacy [31,32].

Clinical studies have highlighted the effectiveness of MN in treating gingival hyperpigmentation, reducing gingival inflammation, as well as in regenerating interdental papillae [33,34,35,36]. In periodontology, MN can accelerate wound healing by stimulating growth factors (PDGF, TGF-α, TGF-β, FGF), angiogenesis, and neo-collagen genesis, with the potential to improve gingival biotype.

The combination of MN + platelet concentrates (e.g., PRF, i-PRF) is mentioned as a promising solution for increasing gingival thickness and tissue regeneration. The combination MN + i-PRF has been tested in clinical studies that demonstrated significant improvement in gingival thickness in patients with thin periodontal phenotype [27,29,37,38], supporting the potential of this technique as a non-surgical and low-morbidity alternative. The combined application of MN and i-PRF synergistic outcomes results in a stable improvement in gingival thickness observed at 1, 3, and 6 months, confirming the clinical relevance of this approach [39].

Microneedles can deliver both small and large molecules, as well as nanoparticles or tissue fluids, ensuring precise local therapy with reduced invasiveness and good patient compliance. In tissue regeneration, MN not only delivers bioactive substances but also directly stimulates cell proliferation and differentiation through induced micro-injuries, supporting angiogenesis and tissue remodeling [40].

In periodontology, microneedles can facilitate the administration of growth factors (e.g., VEGF, PDGF) and platelet concentrates, which play a synergistic role in neovascularization, neo-collagen genesis, and gingival biotype improvement. The MN technology can also integrate smart systems (stimuli-sensitive hydrogels, biodegradable microneedles) for controlled release, opening the way for personalized and safe therapies in periodontal regeneration [21].

PRF represents a three-dimensional fibrin network, rich in platelets and leukocytes, functioning as a biological matrix that promotes tissue healing and periodontal regeneration. The injectable form, i-PRF, was developed by reducing the centrifugation time and speed, resulting in a liquid, fully autologous concentrate that polymerizes slowly in situ [41,42]. It enables a sustained release of growth factors (PDGF, TGF-β, VEGF), which play a crucial role in promoting angiogenesis, fibroblast and osteoblast retrieval, collagen synthesis, and tissue remodeling. Unlike PRP, i-PRF is obtained without the use of anticoagulants, ensuring superior biocompatibility and a lower risk of immune reactions. Due to its injectable consistency and biological properties, i-PRF can be effectively combined with various regenerative approaches, including bone and gingival augmentation, root coverage procedures, gingival thickness enhancement, particularly when associated with MN, and incorporated with specific biomaterials for periodontal regeneration [21,24,25,26,43,44]. In order to obtain i-PRF, a special type of tube is centrifuged at a certain speed for a specific time (700 rpm for 3 min) [24]. The concept of using a lower speed is based on the fact that the componence of the i-PRF is high in platelets, growth factors and leucocytes [24]. An advantage of using this type of PRF is the gel-like consistency that maintains its form for approximately 10–15 min, contributing to the wound-healing process through increased contact and vascularization [44]. When comparing PRP with i-PRF, several studies reported that i-PRF induced a higher cell migration, collagen 1 expression and increased levels of mRNA TGF-β [45]. However, recently, researchers outlined the importance of specific centrifugation protocols, rpm values, centrifugation tube angulation and their role in the cellular accumulation [46]. Miron et al. addressed these issues and optimized the protocol since the initial developed protocols did not provide a very high concentration of platelets and reportedly approximately 23% leucocytes were identified while using the suggested rpm protocol. Further studies revealed that the use of horizontal centrifugation compared to the standard fixed-angle protocols leads to a more increased cell concentration [45]. The results of one study that focused on harvesting a concentrated PRF layer showed that, by using horizontal centrifugation at higher speeds, the cell concentrate and growth factor componence is higher at the buffy coat layer [46].

The local application of bioactive substances via MN increases their bioavailability and therapeutic efficacy, facilitating their penetration into deep periodontal tissues [47]. In addition, MN has been adapted for controlled delivery of immunomodulatory agents such as IL-4 and TGF-β, which can induce macrophage polarization toward the M2 phenotype and the formation of regulatory T cells, promoting a pro-regenerative microenvironment in the periodontium [48].

To our knowledge, the present study is the first one that aims to evaluate the impact of combining these two approaches, i-PRF and MN, in patients diagnosed with periodontal disease. The main focus was to assess the evolution of the active inflammation, the gingival bleeding and the CAL of the patients that underwent the combined protocol (SRP + MN+ i-PRF). The hypothesis that the growth factors encountered in platelet concentrate have a higher impact upon the area where the additional MN was performed may be explained by the fact that the mechanical superficial wound facilitated their contact with the gingival tissue, stimulating the wound-healing cascade controlled by additional growth factors, at the same time. A study performed by Chetana et al. [39] evaluated the MN technique with and without i-PRF for gingival augmentation and their results outlined the positive impact of associating MN to i-PRF, with significant improvement in achieving better tissue thickness. Ozsagir et al. [37] were the first to use the MN on the oral mucosa, providing the first insights regarding the benefits of using a protocol that combines MM and i-PRF in obtaining an improvement in the gingival thickness without a periodontal surgical approach.

Devices such as Dermapen (DermapenWorld, Sydney, Australia) provide control over depth, density, and distribution of micro-perforations, offering a safe and predictable alternative for local delivery of active agents [40].

Our clinical study investigated the short-term periodontal outcome of two regenerative treatment protocols, i-PRF alone (Standard protocol) and i-PRF combined with MN (Combined protocol), in patients with chronic periodontal pockets. Clinical parameters including CAL, BOP, and PI were evaluated over a 6-month period to assess tissue response, inflammation control, and oral hygiene maintenance. Both treatment protocols resulted in significant and sustained improvements in periodontal health parameters. CAL decreased steadily from baseline to 6 months, confirming effective soft tissue reattachment and stabilization. BOP and PI values demonstrated rapid and pronounced reductions within the first month, followed by stable low levels thereafter, reflecting well-controlled inflammation and long-term plaque management. Notably, while statistical comparisons between the Standard and Combined groups did not reveal significant inter-protocol differences, the Combined protocol exhibited slightly greater CAL improvement at 6 months, suggesting that MN may contribute to enhanced early tissue integration and angiogenesis. This modest advantage, though not statistically significant, indicates potential biological benefits worth exploring in long-period follow-ups.

The positive clinical outcome obtained in both groups can be attributed to the well-documented regenerative potential of PRF. Acting as a natural scaffold, PRF gradually releases essential growth factors, including PDGF, TGF-β, and VEGF, which trigger cell proliferation, collagen deposition, and new vessel formation. Its fibrin architecture supports continuous cytokine release, facilitating epithelial repair, angiogenesis, and extracellular matrix organization, ultimately contributing to faster wound closure and enhanced mucosal regeneration [49,50,51].

MN introduces controlled micro-perforations into the gingival tissues, triggering localized inflammation and activating fibroblasts, keratinocytes, and endothelial cells. This controlled trauma increases vascular permeability and facilitates deeper penetration of PRF growth factors into the connective tissue matrix, thus potentially enhancing healing kinetics. Recent randomized controlled trials have demonstrated that MN combined with i-PRF significantly improves gingival thickness and keratinized tissue width in patients with thin periodontal phenotypes [22,29,52].

In the present study, although statistical significance was not reached, the Combined protocol yielded slightly lower median CAL values and more stable attachment gain over time. This trend aligns with histological and in vitro findings showing that PRF stimulates fibroblast proliferation and angiogenesis, while MN promotes the expression of growth factors such as VEGF and FGF, both crucial for early tissue integration [53,54].

The biphasic CAL trajectory observed in both protocols, characterized by a transient increase during the first month followed by a progressive reduction between 3 and 6 months, corresponds with the physiological phases of wound healing. The early rise reflects post-surgical edema and granulation tissue formation, while the subsequent reduction denotes collagen remodeling and reattachment of periodontal fibers. The similar BOP and PI reduction patterns showed that both treatment strategies effectively controlled inflammation and promoted patient adherence to oral hygiene instructions. These results support existing studies, confirming that PRF-assisted therapies can accelerate early soft tissue healing and reduce bleeding tendency by enhancing vascular stabilization and epithelial sealing [55,56].

The clinical relevance of these findings lies in the biological plausibility of MN as an adjunctive procedure for optimizing the regenerative potential of PRF. Although the 6-month results demonstrated only a small numerical advantage for the Combined technique, the trend toward increased CAL improvement suggests that MN may enhance long-term stability of the periodontal attachment. Given that most remodeling occurs within the first 6 months, extended follow-ups at 12 and 24 months are warranted to assess whether early differences translate into measurable long-term attachment gains and bone preservation. Moreover, future studies incorporating histological or imaging-based evaluation (e.g., OCT or Micro-CT) could further clarify the micro-architectural differences between protocols and validate whether MN accelerates collagen maturation or epithelial attachment at the microscopic level.

The primary limitations of this study include the 6-month follow-up period and the lack of histological confirmation of the regenerative outcome. The sample size, although clinically representative, may not be large enough to detect subtle inter-group differences. In addition, while plaque and bleeding were consistently controlled, patient variability in hygiene adherence and systemic factors (e.g., smoking) may have influenced individual responses. Nevertheless, the consistency of improvement across all parameters and the alignment with previously published evidence support the validity of the findings.

## 5. Conclusions

Periodontal disease is a challenging pathology that, besides conventional treatment, can successfully benefit from additional treatment approaches, such as i-PRF, which is a popular technique in dentistry known for its regenerative potential. Its association with MN in the treatment of periodontitis represents an appealing option. In the present study, both the Standard and Combined protocols showed improvement over time, with comparable CAL trajectories and maintained attachment gain at 6 months. The significant and sustained improvement in CAL, BOP, and PI values confirm the regenerative potential of i-PRF in periodontal therapy. The addition of MN showed a modest yet clinically promising enhancement in CAL recovery, likely due to improved early vascularization promotion and improvement of growth factor diffusion. To our knowledge, the present study is the first one that aimed to evaluate the impact of i-PRF combined with MN in patients diagnosed with periodontal disease. Its limitations are related to the number of participants, the short follow-up period and the lack of histological evidence of the cellular population. While short-term differences were limited, the trend suggests that MN may support faster tissue maturation and better long-term attachment stability, hypotheses that warrant prospective 1- and 2-year longitudinal studies for validation. The future perspectives of this minimally invasive, non-surgical treatment approach of periodontal disease focus on improving the methodology and evaluation in order to achieve a complete description of the cellular changes and interactions.

## Figures and Tables

**Table 1:** Summary of demographic characteristics.

Variable	Category	*n*	%
Sex	Female	28	51.85
	Male	26	48.15
Age group (years)	<40	17	31.48
	40–59	36	66.67
	≥60	1	1.85
Environment	Urban	46	85.19
	Rural	8	14.81
Smoking status	Yes	11	20.37
	No	43	79.63

**Table 2:** CAL descriptive statistics by protocol and time point.

Time	Method	*n*	Mean (x)	SD	Median	Q1	Q3	Min	Max	IQR
Baseline	Standard	54	6.30	1.70	6	5	8	4	10	3
Baseline	Combined	54	6.19	1.64	6	5	7	4	10	2
1 month	Standard	54	4.93	1.58	5	4	6	3	9	2
1 month	Combined	54	4.76	1.50	5	4	5	2	10	1
3 months	Standard	54	4.54	1.51	4	3	5	3	9	2
3 months	Combined	54	4.48	1.45	4	3	5	2	9	2
6 months	Standard	54	4.39	1.32	4	3	5	3	8	2
6 months	Combined	54	3.93	1.37	4	3	5	2	8	2

SD: standard deviation.

**Table 3:** Friedman and post hoc Wilcoxon analysis of longitudinal BOP changes.

Comparison	Z	*p*-Value	Effect Size (r)	Interpretation
Baseline vs. 1 month	−5.88	<0.001	0.60	Significant reduction
Baseline vs. 3 months	−6.02	<0.001	0.62	Significant reduction
Baseline vs. 6 months	−5.90	<0.001	0.60	Sustained reduction
3 months vs. 6 months	−0.89	0.374	0.09	No significant change

Z: the standardized value of the Wilcoxon test, indicating the direction and magnitude of the difference between two measurement time points.

## Data Availability

The original contributions presented in this study are included in the article. Further inquiries can be directed to the corresponding authors.

## Abbreviations

The following abbreviations are used in this manuscript:

SDI	sociodemographic index
EFP	European Federation of Periodontology
AAP	American Academy of Periodontology
SRP	scaling and root planing
MN	microneedling
PRF	Platelet-Rich Fibrin
PDGF	Platelet-Derived Growth Factor
TGF-β	Transforming Growth Factor Beta
VEGF	Vascular Endothelial Growth Factor
PPD	periodontal pocket depth
BOP	bleeding on probing
PI	plaque index
CAL	clinical attachment loss
FGF	Fibroblast Growth Factor
TGF-α	Transforming Growth Factor Alpha
OCT	Optical Coherence Tomography
Micro-CT	Micro-Computed Tomography

## References

[1] Wu L., Zhang S., Zhao L., Ren Z., Hu C. (2022). Global, regional, and national burden of periodontitis from 1990 to 2019: Results from the Global Burden of Disease Study 2019. J. Periodontol..

[2] Wu L., Huang C.M., Wang Q., Wei J., Xie L., Hu C.Y. (2025). Burden of severe periodontitis: New insights based on a systematic analysis from the Global Burden of Disease Study 2021. BMC Oral Health.

[3] Hu M., Zhang R., Wang R., Wang Y., Guo J. (2025). Global, regional, and national burden of periodontal diseases from 1990 to 2021 and predictions to 2040: An analysis of the global burden of disease study 2021. Front. Oral Health.

[4] World Health Organization (WHO) Global Status Report on Oral Health 2022. WHO 2022. https://www.who.int/team/noncommunicable-diseases/global-status-report-on-oral-health-2022.

[5] Institute for Quality and Efficiency in Health Care (IQWiG) Overview: Gingivitis and Periodontitis. InformedHealth.org 2023. https://www.ncbi.nlm.nih.gov/books/NBK279593.

[6] Medical News Today What to Know About Gingivitis vs. Periodontitis. Med. News Today. 2024. https://www.medicalnewstoday.com/articles/gingivitis-vs-periodontitis.

[7] Heitz-Mayfield L.J.A. (2024). Conventional diagnostic criteria for periodontal diseases (plaque-induced gingivitis and periodontitis). Periodontol. 2000.

[8] American Academy of Periodontology Glossary of Periodontal Terms. https://members.perio.org/libraries/glossary?ssopc=1.

[9] Texas Periodontal Associates (2021). Gingivitis vs. Periodontitis—Four Stages of Gum Disease. Texas Periodontal Assoc. https://www.texasperiodontal.com/blog/gingivitis-vs-periodontitis-stages-gum-disease/30801.

[10] Chmielewski P., Pilloni A., Cuozzo A., D’Albis G., D’Elia G., Papi P., Marini L. (2025). The 2018 classification of periodontitis: Challenges from Clinical Perspective. Dent. J..

[11] Wang Y., Zhuo L., Yang S., Dong C., Shu P. (2025). Burden of periodontal diseases in young adults. Sci. Rep..

[12] Ravidà A., Troiano G., Qazi M., Saleh M.H.A., Saleh I., Borgnakke W.S., Wang H.-L. (2020). Dose-dependent effect of smoking and smoking cessation on periodontitis-related tooth loss during 10–47 years of periodontal maintenance-A retrospective study in compliant cohort. J. Clin. Periodontol..

[13] Zhang J., Yu J., Dou J., Hu P., Guo Q. (2021). The impact of smoking on subgingival plaque and the development of periodontitis: A Literature Review. Front. Oral Health.

[14] Solomon S.M., Iovan G., Pãsãrin L., Sufaru I.G., Mârţu I., Luchian I., Mârţu M.A., Mârţu S. (2017). Risk predictors in periodontal disease. RJOR.

[15] Hung M., Kelly R., Mohajeri A., Reese L., Badawi S., Frost C., Sevathas T., Lipsky M.S. (2023). factors associated with periodontitis in younger individuals: A scoping review. J. Clin. Med..

[16] Harrel S.K., Wilson T.G., Nunn M.E. (2022). Calculus as a risk factor for periodontal disease. Dent. J..

[17] Kwon T., Lamster I.B., Levin L. (2021). Current concepts in the management of periodontitis. Int. Dent. J..

[18] Harrel S.K., Rethman M.P., Cobb C.M., Sheldon L.N., Sottosanti J.S. (2022). Clinical decision points as guidelines for periodontal therapy. Dimens. Dent. Hyg..

[19] Cobb C.M., Sottosanti J.S. (2021). A re-evaluation of scaling and root planing. J. Periodontol..

[20] Batra P., Dawar A., Miglani S. (2020). Microneedles and nanopatches-based delivery devices in dentistry. Discoveries.

[21] Khan M.U.A., Aslam M.A., Abdullah M.F.B., Gul H., Stojanović G.M., Abdal-Hay A., Hasan A. (2024). Microneedle system for tissue engineering and regenerative medicines: A smart and efficient therapeutic approach. Biofabrication.

[22] Aust M.C., Fernandes J., Kolokythas P., Kaplan H.M., Vogt P.M. (2008). Percutaneous collagen induction therapy: An alternative treatment for scars, wrinkles, and skin laxity. Plast. Reconstr. Surg..

[23] Anegundi R.V., Shenoy S.B., Kaukab S.F., Talwar A. (2022). Platelet concentrates in periodontics: Review of in vitro studies and systematic reviews. J. Oral Med. Oral Surg..

[24] Miron R.J., Choukroun J., Ghanaati S. (2017). Injectable platelet-rich fibrin (i-PRF): Opportunities in regenerative dentistry. Clin. Oral Investig..

[25] Gollapudi M., Bajaj P., Oza R.R. (2022). Injectable platelet-rich fibrin—A revolution in periodontal regeneration. Cureus.

[26] Miron R.J., Gruber R., Farshidfar N., Sculean A., Zhang Y. (2024). Ten years of injectable platelet-rich fibrin. Periodontol. 2000.

[27] Ucer C., Khan R.S. (2023). Alveolar ridge preservation with autologous platelet-rich fibrin (PRF): Case reports and the rationale. Dent. J..

[28] Vesala A.-M., Nacopoulos C. (2025). Microneedling and injectable-platelet rich fibrin for skin rejuvenation and regeneration. Med. Res. Arch..

[29] Yadav A., Tanwar N., Sharma R., Tewari S., Sangwan A. (2024). Comparative evaluation of microneedling vs injectable platelet-rich fibrin in thin periodontal phenotype: A split-mouth clinical randomized controlled trial. Quintessence Int..

[30] Jiang Y., Zhou X., Cheng L., Li M. (2020). The impact of smoking on subgingival microflora: From periodontal health to disease. Front. Microbiol..

[31] Seon-Woo H.S., Kim H.J., Roh J.Y., Park J.H. (2019). Dissolving microneedle systems for oral mucosal delivery of triamcinolone acetonide to treat aphthous stomatitis. Macromol. Res..

[32] Song Y.W., Nam J., Kim J., Lee Y., Choi J., Min H.S., Yang H., Cho Y., Hwang S., Son J. (2025). Hyaluronic acid-based minocycline-loaded dissolving microneedle: Innovation in local minocycline delivery for periodontitis. Carbohydr. Polym..

[33] Mostafa D., Alarawi R., AlHowitiy S., AlKathiri N., Alhussain R., Almohammadi R., Alhussain R. (2022). The effectiveness of microneedling technique using coconut and sesame oils on the severity of gingival inflammation and plaque accumulation: A randomized clinical trial. Clin. Exp. Dent. Res..

[34] Ahuja A., Minz R., Ahuja V., Mishra A., Kumari S. (2022). Evaluation of regenerative potential of locally delivered vitamin C along with microneedling in the treatment of deficient interdental papilla: A clinical study. J. Contemp. Dent. Pract..

[35] Maheshwari A., Tandon S., Lamba A.K., Faraz F., Ansari N. (2025). Management of gingival hyperpigmentation using microneedling with ascorbic acid vs. scalpel technique: A comparative split-mouth study. J. Dent. Res. Dent. Clin. Dent. Prospects..

[36] Qu Y., He Q., Zeng C., Wang L., Ge Z., Liu B., Fan Z. (2025). Microenvironment-regulated dual-layer microneedle patch for promoting periodontal soft and hard tissue regeneration in diabetic periodontitis. Adv. Funct. Mater..

[37] Ozsagir Z.B., Saglam E., Sen Yilmaz B., Choukroun J., Tunali M. (2020). Injectable platelet-rich fibrin and microneedling for gingival augmentation in thin periodontal phenotype: A randomized controlled clinical trial. J. Clin. Periodontol..

[38] Valli Veluri S., Gottumukkala S.N., Penmetsa G.S., Ramesh K., Mohan K.P., Bypalli V., Vundavalli S., Gera D. (2024). Clinical and patient-reported outcomes of periodontal phenotype modification therapy using injectable platelet rich fibrin with microneedling and free gingival grafts: A prospective clinical trial. J. Stomatol. Oral Maxillofac. Surg..

[39] Chetana, Sidharthan S., Dharmarajan G., Iyer S., Poulose M., Guruprasad M., Chordia D. (2024). Evaluation of microneedling with and without injectable-platelet rich fibrin for gingival augmentation in thin gingival phenotype-A randomized clinical trial. J. Oral Biol. Craniofac. Res..

[40] Creighton R.L., Woodrow K.A. (2019). Microneedle-mediated vaccine delivery to the oral mucosa. Adv. Healthc. Mater..

[41] Paccola A.G.L., de Santos T.M.C., Minelo M.C., Garbieri T.F., Sanches M.L.R., Dionísio T.J., de Oliveira R.C., Santos C.F., Buzalaf M.A.R. (2025). Synergistic effects of injectable platelet-rich fibrin and bioactive peptides on dermal fibroblast viability and extracellular matrix gene expression: An in vitro study. Molecules.

[42] Kubesch A., Barbeck M., Al-Maawi S., Orlowska A., Booms P.F., Sader R.A., Miron R.J., Kirkpatrick C.J., Choukroun J., Ghanaati S. (2018). A low-speed centrifugation concept leads to cell accumulation and vascularization of solid platelet-rich fibrin: An experimental study in vivo. Platelets.

[43] Puri K., Khatri M., Bansal M., Kumar A., Rehan M., Gupta A. (2022). A novel injectable platelet-rich fibrin reinforced papilla reconstruction technique. J. Indian Soc. Periodontol..

[44] Żurek J., Niemczyk W., Dominiak M., Niemczyk S., Wiench R., Skaba D. (2024). Gingival Augmentation Using Injectable Platelet-Rich Fibrin (i-PRF)—A Systematic Review of Randomized Controlled Trials. J. Clin. Med..

[45] Miron R.J., Chai J., Zheng S., Feng M., Sculean A., Zhang Y. (2019). A novel method for evaluating and quantifying cell types in platelet rich fibrin and an introduction to horizontal centrifugation. J. Biomed. Mater. Res. A.

[46] Miron R.J., Chai J., Zhang P., Li Y., Wang Y., Mourão C.F.A.B., Sculean A., Fujioka Kobayashi M., Zhang Y. (2019). A novel method for harvesting concentrated platelet-rich fibrin (C-PRF) with a 10-fold increase in platelet and leukocyte yields. Clin. Oral Investig..

[47] Pahade A., Bajaj P., Shirbhate U. (2023). Immunomodulators and their applications in dentistry and periodontics: A comprehensive review. Cureus.

[48] Zhang X., Hasani-Sadrabadi M.M., Zarubova J., Dashtimighadam E., Haghniaz R., Khademhosseini A., Butte M.J., Moshaverinia A., Aghaloo T., Li S. (2022). Immunomodulatory microneedle patch for periodontal tissue regeneration. Matter.

[49] Strauss F.J., Stähli A., Gruber R. (2018). The use of platelet-rich fibrin to enhance the outcomes of implant therapy: A systematic review. Clin. Oral Implant. Res..

[50] Ehrenfest D.M., Rasmusson L., Albrektsson T. (2009). Classification of platelet concentrates: From pure platelet-rich plasma (P-PRP) to leucocyte-and platelet-rich fibrin (L-PRF). Trends Biotechnol..

[51] Ghanaati S., Herrera-Vizcaino C., Al-Maawi S., Lorenz J., Miron R.J., Nelson K., Schwarz F., Choukroun J., Sader R. (2018). Fifteen years of platelet rich fibrin in dentistry and oromaxillofacial surgery: How high is the level of scientific evidence?. J. Oral Implantol..

[52] Chandrashekar B.S., Sriram R., Mysore R., Bhaskar S., Shetty A. (2014). Evaluation of microneedling fractional radiofrequency device for treatment of acne scars. J. Cutan. Aesthet. Surg..

[53] Rattanawonsakul K., Seleiro G., Workman V., Claeyssens F., Bolt R., Seemaung P., Hearnden V. (2025). The effect of liquid platelet-rich fibrin on oral cells and tissue engineered oral mucosa models in vitro. Sci. Rep..

[54] dos Santos Guimarães L.H., Pereira Neto A.R.L., de Oliveira T.L., da Silva Kataoka S.M., de Jesus Viana Pinheiro J., de Melo Alves Júnior S. (2024). Platelet-rich fibrin stimulates the proliferation and expression of proteins related to survival, adhesion, and angiogenesis in gingival fibroblasts cultured on a titanium nano-hydroxyapatite-treated surface. J. Oral Biosci..

[55] Del Corso M., Vervelle A., Simonpieri A., Jimbo R., Inchingolo F., Sammartino G., Dohan Ehrenfest D.M. (2012). Current knowledge and perspectives for the use of platelet-rich plasma (PRP) and platelet-rich fibrin (PRF) in oral and maxillofacial surgery part 1: Periodontal and dentoalveolar surgery. Curr. Pharm. Biotechnol..

[56] Neophytou C., Dorda M., Balouri A., Dimitriadou P., Neofytou A.-M., Batas L. (2025). The role of platelet-rich fibrin (PRF) in periodontal plastic surgery: A contemporary review of evidence-based applications. Int. J. Clin. Stud. Med. Case Rep..

